# Molecular identification of archaic bones as a native Korean black bear: implications for the ongoing bear restoration program

**DOI:** 10.1080/19768354.2022.2112755

**Published:** 2022-09-20

**Authors:** Jee Yun Hyun, Tae-Wook Kim, Puneet Pandey, Kyung Seok Kim, Seung-Jun Jeong, Jae-Ku Kang, Dal-Yong Kong, Seung-Ho Jung, Ho-Kweon Jeong, Sang-Hoon Han, Sang-Hyun Han, Hang Lee

**Affiliations:** a Conservation Genome Resource Bank for Korean Wildlife (CGRB) and Research Institute for Veterinary Science, College of Veterinary Medicine, Seoul National University, Seoul, Republic of Korea; b National Institute of Biological Resources, Incheon, Republic of Korea; c Habitat Conservation Division, Korea National Park Research Institute, Korea National Park Service, Yeongju, Republic of Korea; d ENPROTEC India Foundation, Uttar Pradesh, India; e Department of Pediatrics-Nutrition, Baylor College of Medicine, Houston, USA; f Korea National Park Service, Wonju, Republic of Korea; g International Cooperation Division, Cultural Heritage Administration, Daejeon, Republic of Korea; h National Research Institute of Cultural Heritage, Cultural Heritage Administration, Daejeon, Republic of Korea; i Ecosystem of the Korean Peninsula Research Institute, Jeongseon, Republic of Korea; j Inter-Korea Wildlife Institute, Inje, Republic of Korea

**Keywords:** Asian black bear, radiocarbon dating, molecular identification, cave bone

## Abstract

The genetic investigation of the archeological or museum samples, including endangered species, provides vital information necessary to plan, implement, and revisit conservation strategies. In South Korea, the Asian black bear went almost extinct in wild by 2002, without leaving any authentic specimens representing the native population. Recently researchers found a set of animal bones in a natural cave in Mt. Taebaek (South Korea), suspected to be of a bear. In the present study, we undertook a molecular investigation and radiocarbon dating to establish the species’ identity, phylogenetic position, and approximate age of the recovered specimen. The genetic investigation (CytB, COI, D-loop, SRY, and ZFX-ZFY) identified the sample as a male Asian black bear with close phylogenetic affinity with Northeast Asian bears. Radiocarbon dating estimated the bones to be aged 1800–1942 calAD. These findings indicate that the bone specimens found in the natural cave in Mt. Taebaek were from an individual that naturally inhabited South Korea long before the importing of farm bears (the 1980s) and initiation of wild population restoration (2004). The present study provides the first genetic information record of the native South Korean black bear. Our findings reaffirm the appropriateness of the ongoing bear restoration program in South Korea, with the reintroduction of individuals from North Korea and Russia.

## Introduction

Ancient or old DNA-based studies are useful in understanding the phylogenetic relationship between extinct and extant species. Museum sample genetic analysis was found to be useful in establishing a phylogenetic relationship between extinct quagga and extant mountain zebra (Higuchi et al. 1984). Based on the mitochondrial DNA analysis of Holocene sub-fossil giant panda and modern panda, Sheng et al. (2018) concluded that extant panda populations do not suffer any immediate threat from the perspective of genetic evolutionary potential. Ancient DNA analysis was also found useful to elucidate the evolutionary history of Yakutian brown bears and Japanese otters (Park et al. 2019; Rey-Iglesia et al. 2019). Archeological genetic studies are difficult to perform as old fossilized samples yield low-quality DNA with high chances of environmental contamination (Rohland and Hofreiter 2007). These studies require dedicated laboratory facilities, skilled researchers, and efficient protocols for DNA extraction and PCR.

Asian black bear is included in the endangered species list of the South Korean Ministry of Environment. In South Korea, Asian black bears can be categorized into three groups – native, farmed, and reintroduced. Bear farms were established in the 1980s to support local livelihood (MOE 2005; Lee et al. 2013; Jang 2014). The genetic ancestry of farmed black bears is ambiguous and unknown (Kim et al. 2012). The native Asian black bear population of South Korea was predicted less than 5 by the early twenty-first century due to excessive persecution and habitat loss. The South Korean government initiated the Asian black bear population restoration project (Mt. Jiri), in 2004, using individuals imported from North Korea, China, and Russia (Lee and Jeong 2009; Kim et al. 2012). According to the International Union for Conservation of Nature (IUCN) guidelines for re-introductions, the population restoration of endangered species should be preceded by a phylogenetic examination of the native population to ensure the selection of candidates from a genetically similar population or subspecies (IUCN 1998). Hong (2005) and Kim et al. (2011) conducted a phylogenetic study on Asian black bears, including the Ussuri subspecies, and found that candidate populations, for the South Korean Asian black bear restoration project, were appropriate. However, their examination does not include any native South Korean Asian black bear specimen.

Asian black bears are divided into seven subspecies based on their geographical distribution (Won 1968; Smith and Xie 2008; Ohdachi et al. 2015) and are distributed in a wide area of East Asia, Southeast Asia, and South Asia. Among these, the Asian black bear populations in the Korean Peninsula-Northeast China–Russia Far East Asian region are considered and classified as subspecies *U. t. ussuricus* (Won 1968; Smith and Xie 2008; Ohdachi et al. 2015). In South Korea, there exists confusion about the subspecies nomenclature of native Asian black bears with the use of the two scientific names – *U. t. ussuricus* (major use) and *U. t. wulsini* (rare use) (Jo et al. 2018). This confusion might have arisen because of the similar pronunciation between Chihli of Hebei (China), the type locality of *U. t. ussuricus*, and Mt. Jiri (South Korea) (Cultural Heritage Administration 2016; Jo et al. 2018). Thus, a re-evaluation of the phylogenetic position of the native South Korean black bears is required to clarify the controversy.

In 2016, a set of bones was found in a cave in South Korea, presumed to be Ursidae based on morphology. In Korea, most of the bear bones excavated from natural caves or prehistoric sites belong to brown bears, and the remains and fossils of Asian black bears have rarely been found (Won 1967; Jeong 2018; Jo et al. 2018). The discovery of these bones could offset the lack of sample availability, and using these samples, we developed the following hypotheses: (1) Recovered bones belong to the Asian black bear (*Ursus thibetanus*), (2) Asian black bear in hypothesis-1 is an animal that naturally lived in the wild in South Korea and is not a bear (or descendant) of a domestic farm, introduced from abroad in the 1980s. (3) The South Korean Asian black bear population had an active genetic exchange with other populations in Northeast Asia (North Korea, Northeastern China, and Far East Russia) and represent the same clade in a phylogenetic tree – Northeast Asia clade or *Ursus thibetanus ussuricus.* We undertook a scientific study using molecular techniques and radiocarbon dating to test the above hypothesis. We examined the recovered sample to determine – (1) species of origin (black or brown bear), (2) approximate age, and (3) phylogenetic position.

## Material and methods

### Samples and DNA extraction

**Figure 1.**
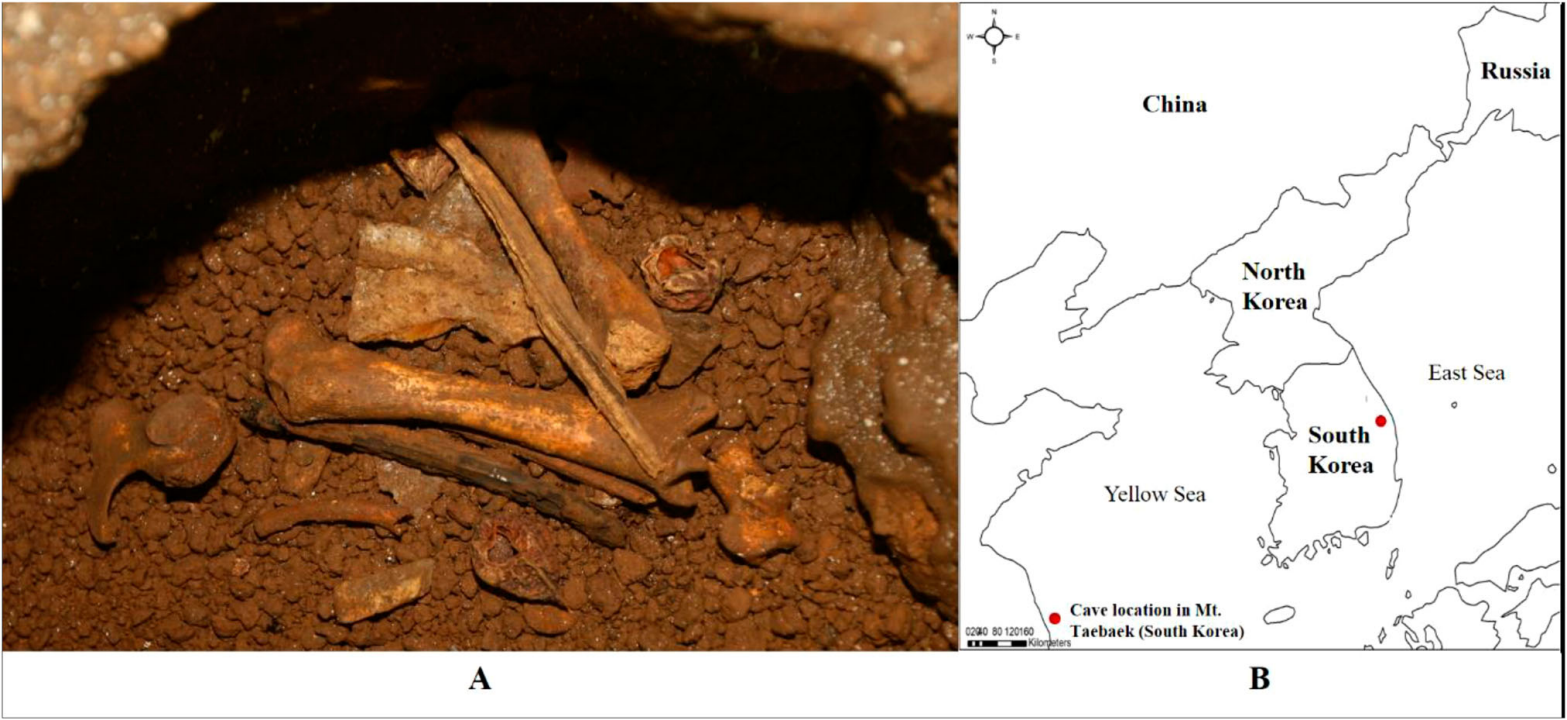
The animal bone specimens from a natural limestone cave in Mt. Taebaek in South Korea that we used in this study – (A) bone specimen and (B) cave location (marked with a red dot).

Samples were obtained from a set of animal bones found in a natural limestone cave in the Mt. Taebaek area, Samcheok City, Gangwon Province, Korea (N37.0451, E129.0936, 700 m) (Figure 1). These bones were initially assumed to be of a carnivore (Jeong 2016) and were later identified as bear bones based on the morphological characterization (bone size and shape) by an expert (Swenson et al. 2007; Huber and van Manen 2019). The bear bone specimens have been stored in the Natural Heritage Center (Geology) of Korea and registered with specimen number NHCG a10953.

The DNA extraction was performed in an isolated, sterile facility dedicated to ancient and museum samples at the Seoul National University College of Veterinary Medicine (South Korea). We selected the thigh bone for DNA extraction. The bone was cleaned, sterilized, and punctured using a drill, and bone powder was collected from the bone marrow region (Rohland and Hofreiter 2007). DNA was extracted from the bone powder using the QIAamp DNA Micro Kit (Qiagen, Germany) following the supplied protocol for bone DNA extraction. Negative controls were included in every step to monitor contamination.

### Polymerase chain reaction (PCR)

For the molecular analysis of the bone samples from the cave, fragments of the D-loop region (620 bp), cytochrome b gene (CytB, 697 bp), and cytochrome oxidase subunit I gene (COI, 631 bp) in mtDNA, ZFX-ZFY, and SRY gene in nDNA were PCR amplified. CytB and COI genes are commonly used for species identification and mtDNA D-loop for phylogenetic analysis of mammals (Tobe et al. 2009; Yasukochi et al. 2009; Kartavtsev 2011; Kartavtsev 2013; Shen et al. 2013; Lee et al. 2016). According to the morphological characteristics of the bones, we used bear-specific primers. For efficient PCR amplification of archaeological DNA, we used primers that amplified a short target sequence. All primers except for the URL2/URH2 primer pair (Uchiyama 1998) against the D-loop region were newly constructed using the published Asian black bear DNA sequence (GenBank Accession No. EF681884.1, AM748310, AM941050) (Table 1). Primers were designed using Primer3 software (Untergasser et al. 2012).

**Table 1.** Primer information in this study.

Primer	Sequence information	Target	Reference
URL2	5′-CTGTTTAAACTATTCCCTGGTACAT-3′	D-loop	Uchiyama (1998)
URH2	5′-GCCTGGTGATCAAGCTCCCGGAC-3′	D-loop	Uchiyama (1998)
UR1	5′-GCG TATAGTCTGTAAGCAT-3′	D-loop	This study
UR2	5′-GCTGATAGTCATTAGTCCATC-3′	D-loop	This study
UR4	5′-CTAGATTCCAATCCTACTAACC-3′	D-loop	This study
UR5	5′-CGTTCGATTCAGCGGTATTT-3′	D-loop	This study
CytBF	5′-GACGCGACTACAGCCTTTTCAT-3′	CYTB	This study
CytB218R	5′-CCTATGAATGCGGTGGCTATTC-3′	CYTB	This study
CytB770R	5′-CCTCGTTGTTTGGATGTGTGTAG-3′	CYTB	This study
COI627F	5′-GCTCTCAGCCTTTTGATTCGTG-3′	COI	This study
COI157F	5′-TAATCACGGCAGTGCTTCTTCT-3′	COI	This study
COIR	5′-GGGTGTCCGAAGAATCAAAAC-3′	COI	This study
SRY5F	5′-CCCCAACCCCTTTTCTTTTA-3′	SRY	This study
SRY5R	5′-GTTCTGGCCGCTGTCTCTAC-3′	SRY	This study
SRYF	5′-ATGCACCGACAGAAATACCC-3′	SRY	This study
SRYR	5′-TTAGCTGGTCCTCCATTCCTG-3′	SRY	This study
SRY3F	5′-CCGAGAAACCTTGGCTACAC-3′	SRY	This study
SY3R	5′-AAGCAGCCATAAACCCAGAC-3′	SRY	This study
ZFF	5′-AGGGCACATGAGTTCCACAG-3′	ZFX, ZFY	This study
ZFR1	5′-TTTATCCCAGGAAATCATTCATG-3′	ZFX, ZFY	This study
ZFR2	5′-TTTTTATCCCAGGAAATCATTCA-3′	ZFX, ZFY	This study

The PCR (Takara TP600) cycle conditions for mtDNA fragments were as follows: 5 min initial denaturation at 94°C; 50 cycles of 45 s at 94°C, 45 s at 54°C, and 45 s at 72°C; and a final extension for 5 min at 72°C. Similarly, PCR amplifications were performed for SRY and ZFX-ZFY genes using the following conditions: 5 min initial denaturation at 94°C; 40 cycles of 45 s at 94°C, 45 s at 58°C for SRY and at 56°C for ZFX-ZFY, and 45 s at 72 °C; and a final extension for 10 min at 72°C. PCR negative was included in each experiment to monitor contamination. Post amplification, amplified products were resolved on 1.5% agarose gel.

### DNA sequencing

The amplified PCR products were sequenced using a Big Dye version 3.1 cycle sequencing kit and ABI Prism 3730XL DNA analyzer (Applied Biosystems, Foster City, CA, USA). The sequences determined in this study were used to identify the species by comparing their similarity with sequences reported in the NCBI nucleotide database through BLAST searches (Supplementary information 1).

For sex identification, the DNA sequences of the bone specimens were compared to those of male and female Asian black bears as controls. Sex discrimination was performed based on the presence or absence of the SRY gene and homozygosity/heterozygosity of the ZFX-ZFY gene.

### Phylogenetic analysis

The 620 bp sequence of the D-loop region was used for phylogenetic analysis as in previous studies (Kim et al. 2011). For the phylogenetic analysis, the corresponding sequence of Asian black bears from China, Russia, Japan, and Southeast Asia (Accession Nos.: HM135178-135193 and EU264496-264527) were obtained from NCBI GenBank. The American black bear (*Ursus americanus*) (Accession Nos.: HE657194, HE657196) was used as the outgroup. Geneious 5.0.4 (Kearse et al. 2012) was used for multiple sequence alignment, and a phylogenetic tree was constructed using IQtree 1.5.6 with an ultrafast bootstrap of 1000 replicates (Minh et al. 2013; Hoang et al. 2018; Kalyaanamoorthy et al. 2017). The best-fit model was chosen based on the Bayesian information criterion (BIC) (Nguyen et al. 2015; Hoang et al. 2018; Kalyaanamoorthy et al. 2017). The maximum-likelihood (ML) consensus trees were illustrated using FigTree v 1.4.3 (http://tree.bio.ed.ac.uk/software/figtree/).

### Radiocarbon dating

The age of the bone specimens was determined using the radiocarbon dating method proposed by the Korea Institute of Geoscience and Mineral Resources (KIGAM), Daejeon, South Korea. The estimated age calculations used Libby’s half-life (5568 years) (Arnold and Libby 1949; Stuiver and Polach 1977), which is relative to the year 1950.

The bone powder was used for radiocarbon dating. The foreign particles were removed from the bone and collagen was extracted and gelatinized (Kim et al. 2010). The whole process includes the following steps – (1) the sample was added to 0.5 M HCl for 1 h at room temperature and washed to be neutralized, (2) the sample was added to 0.1 M NaOH solution for 1 h at room temperature and washed to neutral pH, (3) the sample was added to 0.5 M HCl for 30 min at room temperature and neutralized, (4) the sample was added to HCl solution (pH 3) for 12 h at 70° to proceed with gelatinization. Then, the sample was collected that passed through the 2.7 μm filter. Finally, using a Centriprep ultrafilter, gelatin with a molecular weight of ≥30,000 Da was collected (Kim et al. 2010) and freeze-dried using OPR-FDG-120 lyophilizer (OPERON). In the processed samples, ^12^C, ^13^C, ^14^C, ^13^C/^12^C, and ^14^C/^12^C were measured using AMS (Accelerator Mass Spectrometer). The radiocarbon age and δ^13^C were calculated according to the method proposed by Stuiver and Polach (1977). Then the calendar age was analyzed using calibration curves of IntCal13 (Reimer et al. 2013) and Korean Tree Ring (Hong et al. 2013) with Bayesian statistics (Ramsey 2009).

## Results

### Species identification: cave bones belong to a male Asian black bear

The partial nucleotide sequences of the CytB gene (697 bp), and COI gene (631 bp) were determined from the mtDNA fragments amplified from the thigh bone. Results of the BLAST search revealed that the partial mtDNA gene sequences from the unknown animal bone were of the Asian black bear, *Ursus thibetanus* (Supplementary information 1). The COI and CytB sequences were 100% identical to GenBank Accession Nos. EF6667005 and EU573174, respectively (Supplementary information 1). All mitochondrial gene sequences used in this study matched 100% to the sequences of Asian black bears (i.e. CGRB54, Ducksung17, Jangkang21, and Songwon), which had been reintroduced to Mt. Jiri from North Korea. Therefore, the investigated animal bones belong to the Asian black bear, *U. t. ussuricus*.

**Figure 2.**
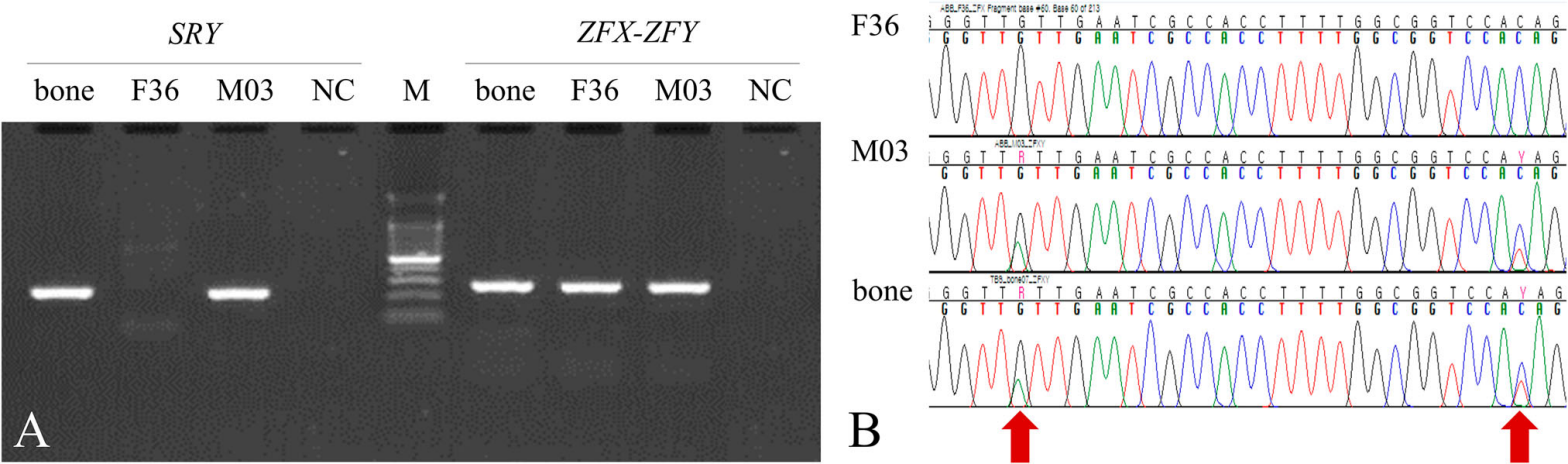
Molecular sexing for the animal bone DNA. (A) PCR amplification for male-specific *SRY* and sex-chromosome-linked *ZFX-ZFY* genes. (B) Multiple alignments for DNA sequences of *ZFX-ZFY* amplified in the PCR test (A). Bone indicates the DNA sample of animal bone found in the Taebaeksan area. F36 and M03 are female and male bears reintroduced from China and Russia, respectively. NC is the negative control for PCR amplification and M is the DNA size marker, 100 bp DNA Ladder.

In molecular sex determination tests, the fragment of the SRY gene (196 bp) was successfully amplified (Figure 2) from the DNA extracted from the bear bone, and the nucleotide sequence was consistent with the sequences of the SRY gene previously reported for Ursidae. In addition, the amplified ZFX-ZFY (288 bp) gene sequence showed heterozygosity (Figure 2), suggesting that this investigated sample was a male Asian black bear.

### Radiocarbon dating: identified male Asian black bear that lived before the Korean War

**Figure 3.**
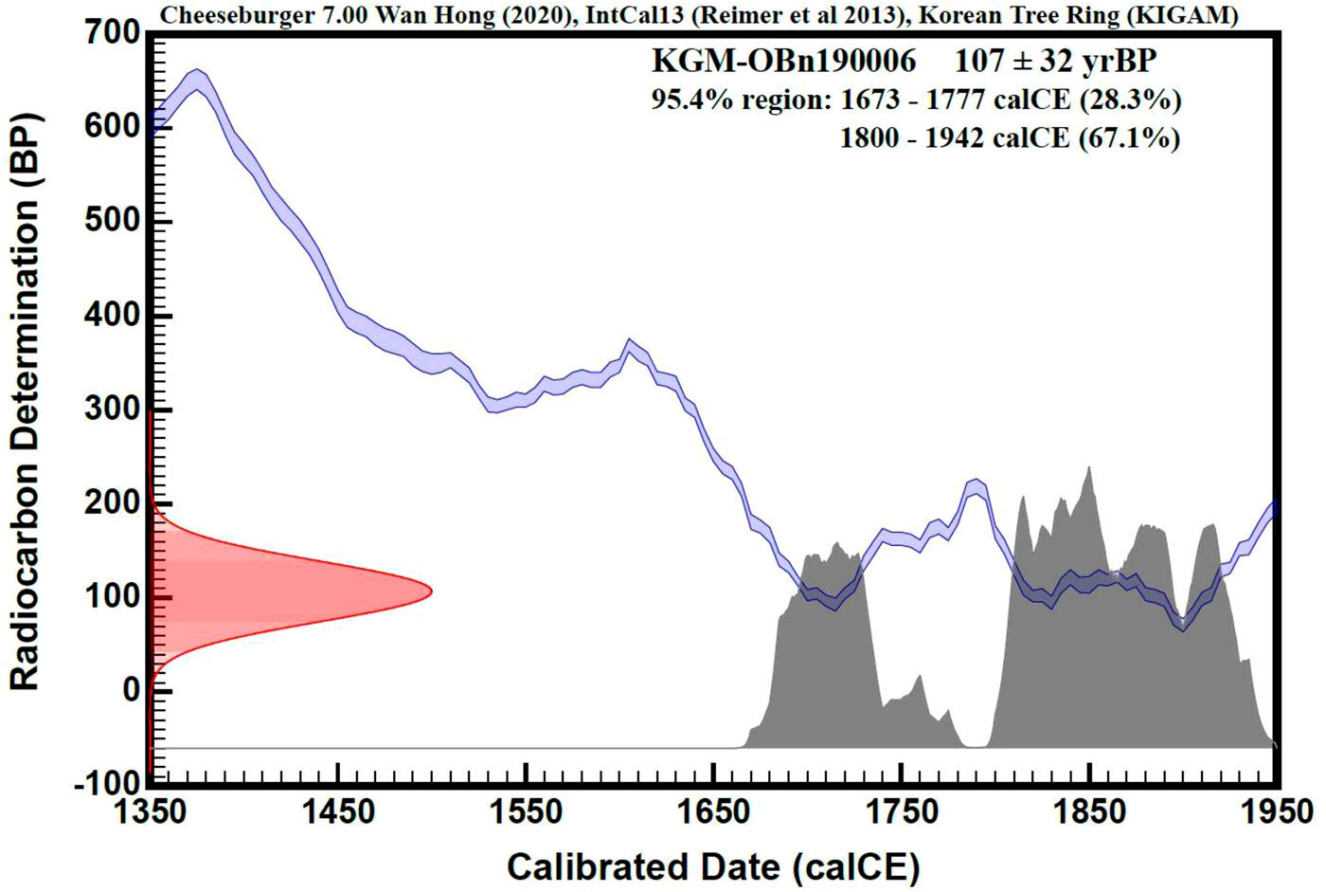
Age dating graph. Radiocarbon determination dates (^14^C age) are calculated by Libby’s half-life, based on the ratio of ^14^C/^12^C (ordinate). The red color indicates the radiocarbon determination (BP) results of the bone sample. The Abscissa shows calibrated date (calCE = calAD) which is adjusted data of radiocarbon determination using calibration curves (blue line) of IntCal13 and Korean tree ring. The Grey color indicates calibrated age dating of bear specimens in the 95.4% region.

Radiocarbon dating based on the ratio of the carbon isotopes ^12^C and ^14^C produced estimates of 1843 ± 32 yrsBP. After the carbon date was adjusted to calibrated date using IntCal13 and Korean Tree Ring calibration curves, a range of 1800–1942 calCE (median 1871) was indicated at a 67.1% probability (Figure 3). Radiocarbon dating was highly reliable as our inferred sample age range (1800–1942) lies within the 95% accuracy range (Figure 3). Within the age accuracy range of 95.4%, two distinct age ranges were observed 1673–1777 (28.3% probability) and 1800–1942 (67.1% probability), thus age was determined 1800–1942 calCE. In other words, the bones found in the cave in the Mt. Taebaek area were estimated to be from a bear that lived on the Korean peninsula 80–220 years ago. The results of the age analysis methods suggested that the age of this sample was estimated to be 1800–1942 AD (Mid-point to late 1800s), i.e. approximately 150 years ago. This period corresponds to the late Joseon Dynasty (nineteenth century) or early twentieth century, before the Korean War in 1950.

### Phylogenetic position and conservation of the Asian black bear native to South Korea

**Figure 4.**
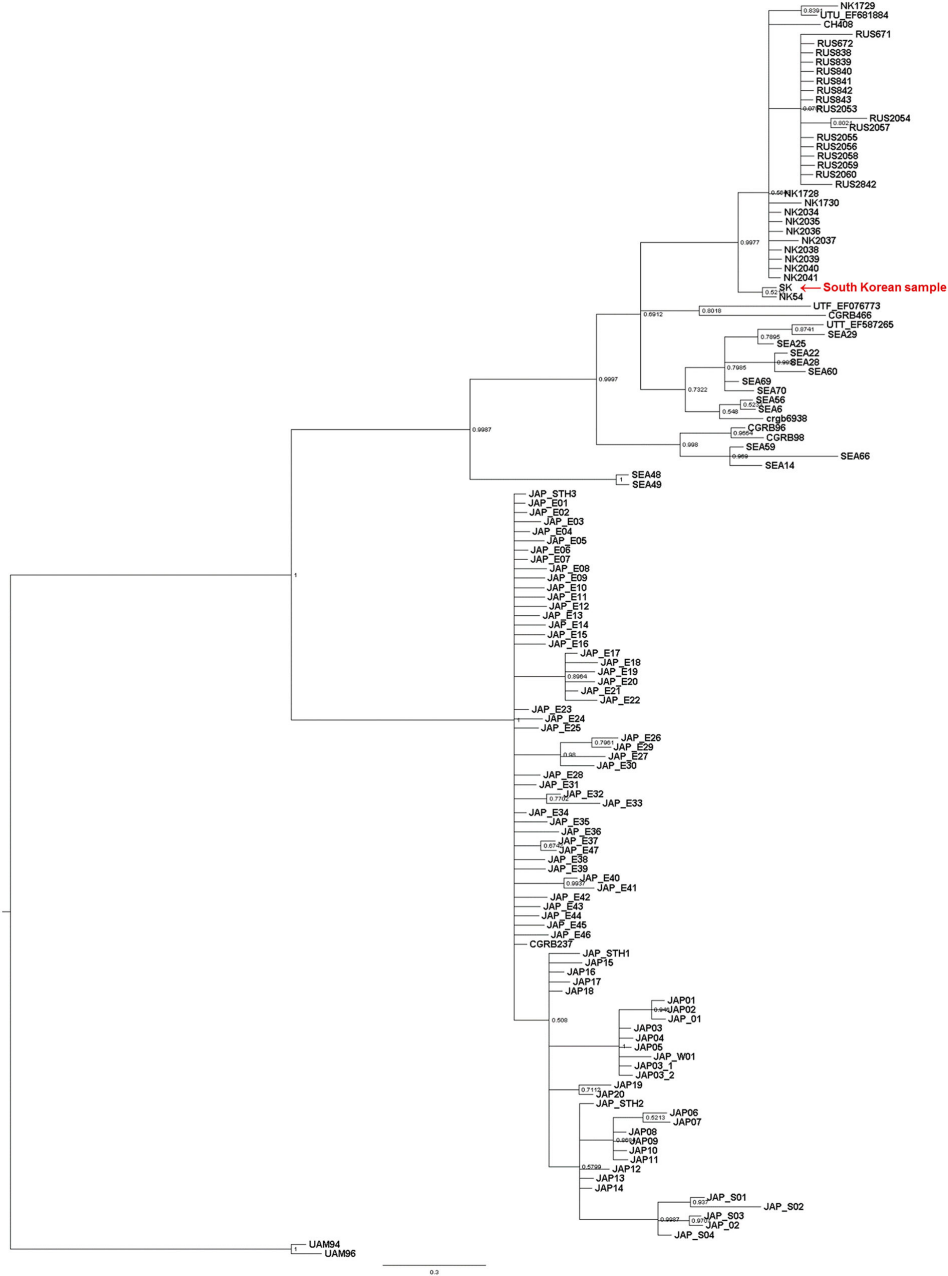
Phylogenetic tree of the Asiatic black bear using a 620 bp fragment of the mitochondrial D-loop region.

Analysis of the phylogenetic relationships using the D-loop region of mtDNA showed that this South Korean bear was grouped with Asian black bears from North Korea, China, and Russia and that it was distinct from black bears from Southeast Asia and Japan (Figure 4). Due to a single representative sample for each northeast China and South Korea in our study, we recommend having further detailed bear phylogeny in the future with additional representative samples for under-sampled northeast China and South Korea.

## Discussion

Asian Black bears inhabited much of the Korean peninsula until the early decades of twentieth century and their population plummeted due to habitat loss, capture, and hunting. A wild bear was reportedly poached in Mt. Seorak in 1983, since then, there had been no formal reports of wild bears in South Korea until 2000. In 2000, the activities of a black bear in Mt. Jiri were filmed by a broadcaster's cameras, but the number of Asian black bears in the wild has been very low, and these bears have been considered to be almost extirpated (KNPS 2004; Yang 2008). Since 2004, the reintroduction of Asian black bears from Russia, China, and North Korea has been carried out in Mt. Jiri to restore the Asian black bear population (MOE 2005; Lee and Jeong 2009; SRTI 2018). Additionally, Asian black bears were recently confirmed to inhabit the demilitarized zone (DMZ) via the use of unmanned sensor cameras (MOE 2016; MOE 2019).

Understanding of molecular affinity (species status and phylogenetic position) of archeological samples is essential to maintain the utility in species conservation and restoration. Black bears represented the Korean Peninsula in all previous genetic studies were from North Korea due to a lack of authentic native South Korean specimens. Phylogenetic analysis confirmed the species (Asian black bear) and subspecies identity (Ussuri spp) of recovered bones. The radiocarbon dating of specimens suggested that the bear bones found in the cave did not originate from bears imported into South Korea between 1981 and 1985 for bear farming. This confirms that the bones found and investigated in the present study originated from a bear belonging to the native South Korean population of Asian black bears. The finding has academic significance as no prior specimen has been found derived from an Asian black bear that lived in South Korea before the 1950s.

The phylogenetic results presented here indicate that the native South Korean, North Korean, and Russian Asian black bears belong to the same genetic group. Therefore, it would be the best strategy to reintroduce individuals from Northeast Asia, including regions such as North Korea, Russia, and northeast China. The present study is supported by previous studies (Hong 2005; Kim et al. 2011; Wu et al. 2015) suggesting that the conservation unit of the Asian black bear group is composed of black bear populations of the Korean peninsula (with North Korean samples), Russian Primorskiy region, and northeastern China. Furthermore, the results of our study indicate that the selection of individuals from Far Eastern Russia, northeast China, and North Korea for the ongoing bear reintroduction project (Lee and Jeong 2009; Kim et al. 2011; SRTI 2018) was the appropriate choice, in that they are from Asian black bear subspecies that share the same genetic group with the native South Korean black bears.

## Conclusion

The unknown animal bones found in the Mt. Taebaek limestone cave were identified to be the remains of a wild male Asian black bear (*Ussuri subspecies*) that inhabited the Korean Peninsula in the nineteenth century or early twentieth century. Moreover, the findings reaffirm the management decision of selecting Northeast Asian black bears as the source population for the reintroduction project in South Korea.

In wildlife conservation, it is important to preserve a sufficient population size to ensure that the species does not become endangered, but it is also critical to maintaining the unique biological characteristics of the population that has accumulated for an extended period in one area (Lee et al. 2021). The bear bones identified in this study are precious specimens that can clarify the biological features inherent to the Asian black bears that inhabited South Korea. In the future, information on the osteological, morphological, and genetic characteristics of these samples will be used not only to describe the features of the native population of Asian black bears in South Korea but also to contribute to understanding the evolution and regional differences in the Northeast Asian black bear populations, including those from the Korean peninsula.

## Supplementary Material

Supplemental Material
