# Supplementary material for: Molecular identification of archaic bones as a native Korean black bear: implications for the ongoing bear restoration program
Source: Anim Cells Syst (Seoul). 2022 Sep 20;26(5):214–22. doi: 10.1080/19768354.2022.2112755 (PMC9586619; doi:10.1080/19768354.2022.2112755)
Supplement: Supplemental Material [file TACS_A_2112755_SM7561.docx]

Supplimenatary information 1: Results of NCBI – BLAST search for three mitochondrial DNA sequences (CytB and COI)

1. Cytochrome B gene (697 base pair)


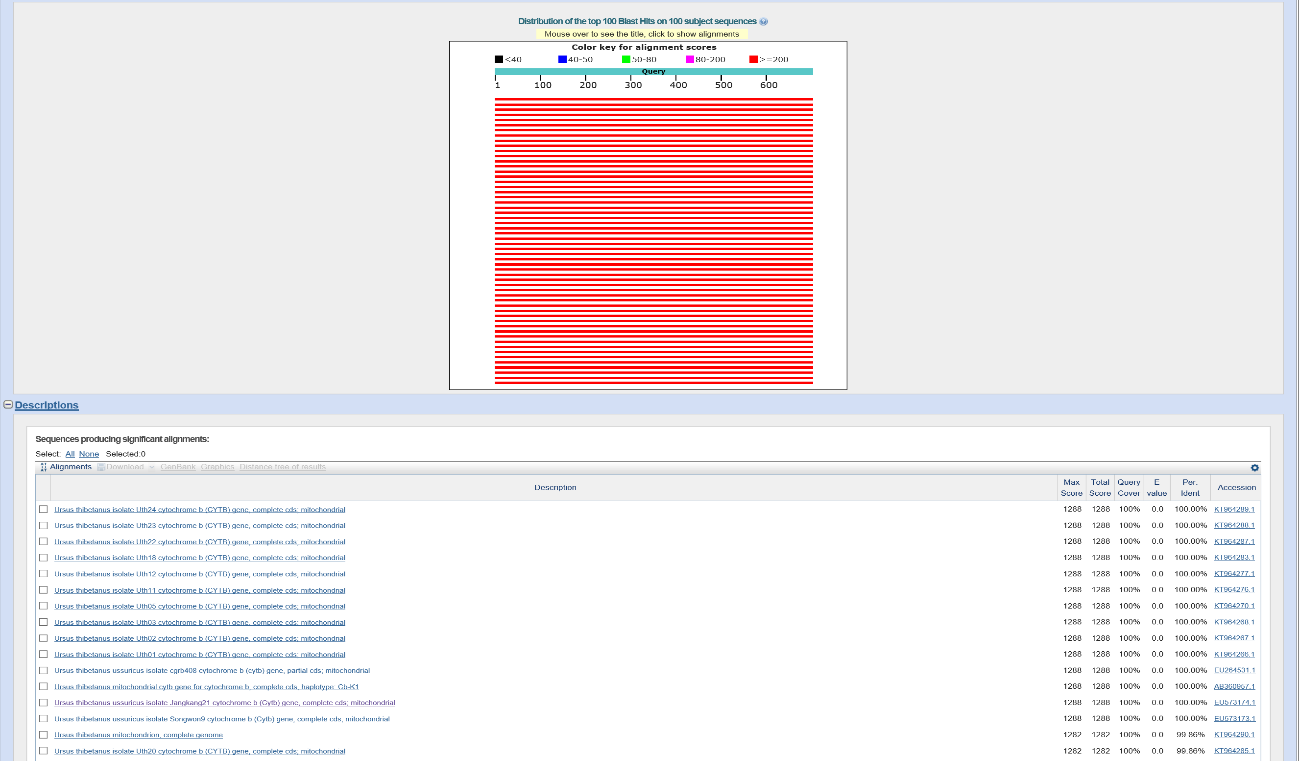


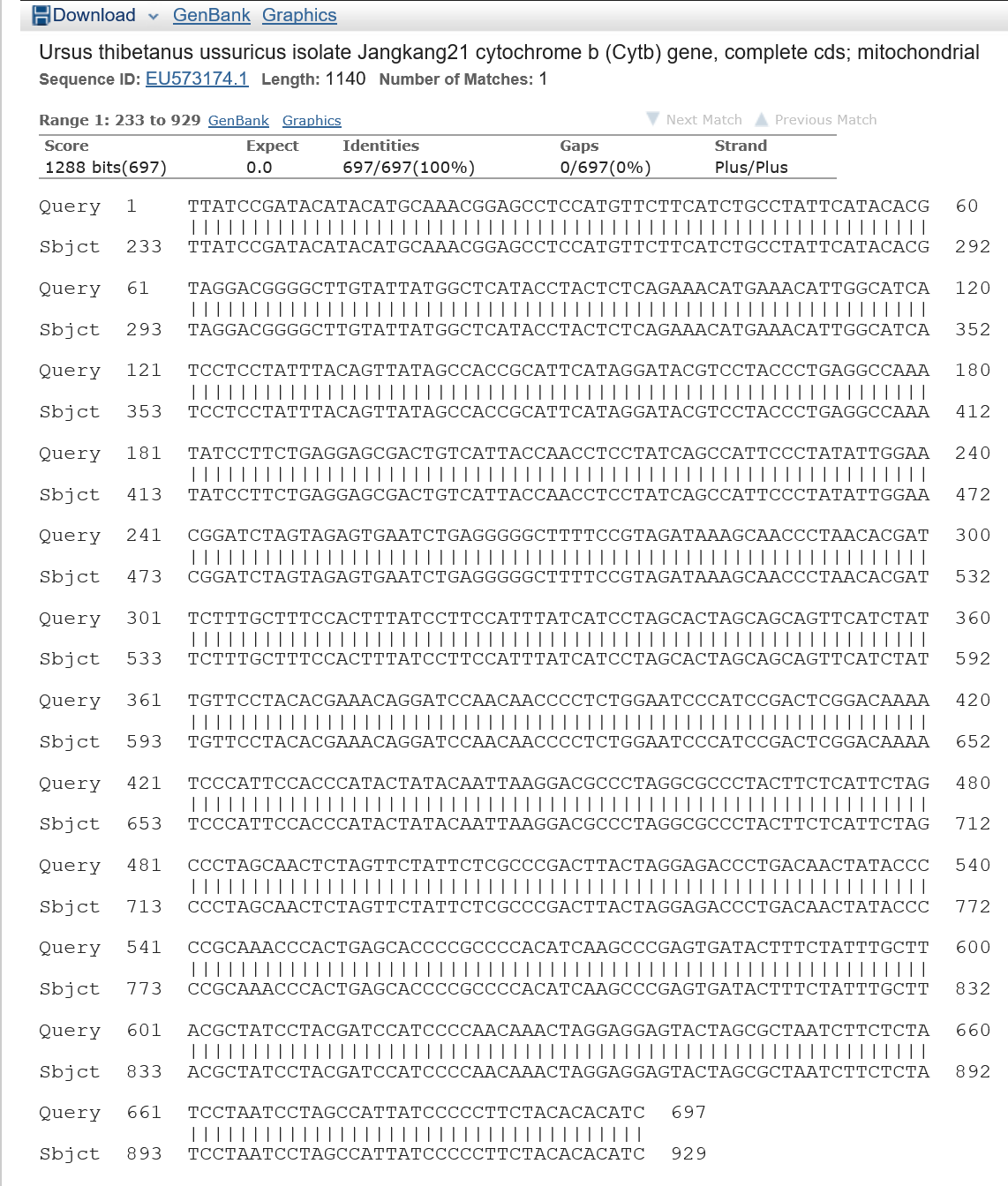


Result:

- 100% similarity with Asiatic black bear (*Ursus thibetanus*)

- 697/697: KT964266-68, KT964270, KT964276-77, KT964283, KT964287-89,

EU264531(cgrb408), AB360957(Cb-K1, Russia and North Korea), EU5713174(Jangkang21, North Korea), EU5713173(Songwon9, North Korea)

1. Cytochrome oxidase I (631 base pair)


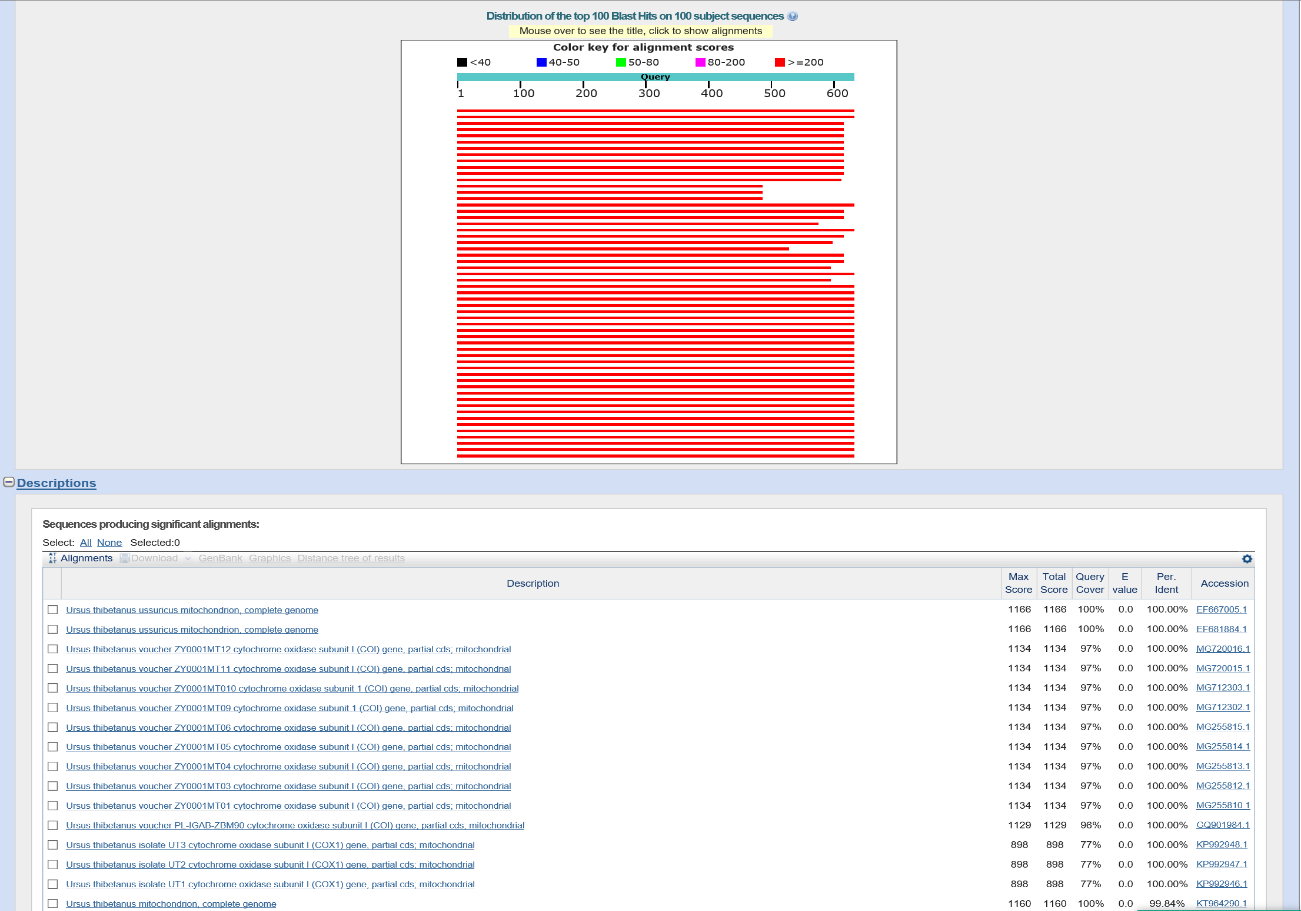


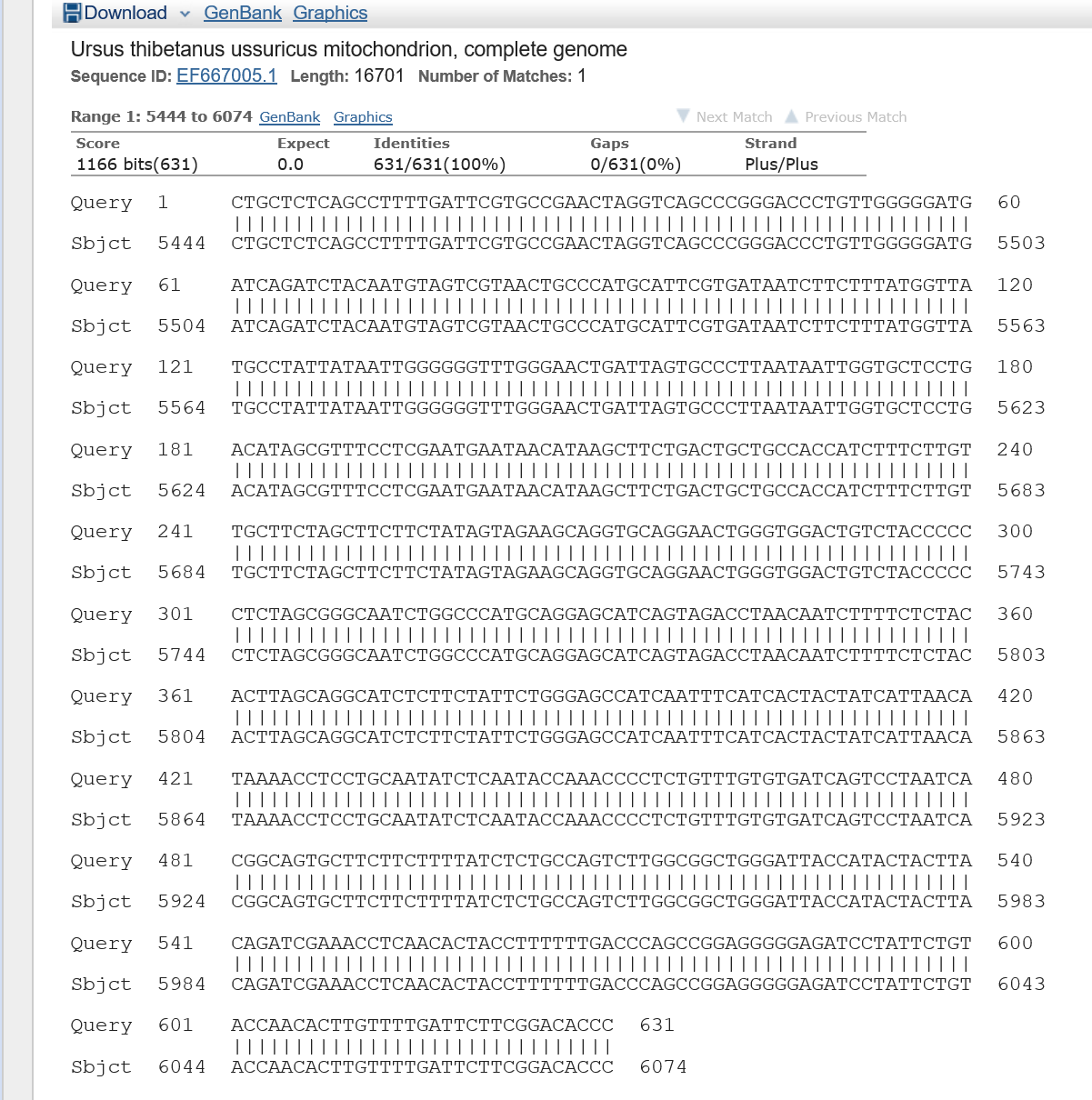


Result:

- 100% similarity with Asiatic black bear (*Ursus thibetanus*)

- 631/631: EF667005, EF681884

- 614/614: MG720015-16, MG712302-03, MG255810, MG255812-15
